# Primary Focal Segmental Glomerulosclerosis among Patients with Glomerular Disease Undergoing Kidney Biopsy in a Tertiary Care Centre: A Descriptive Cross-sectional Study

**DOI:** 10.31729/jnma.8039

**Published:** 2023-02-28

**Authors:** Bikash Khatri, Anil Baral, Suresh Maharjan, Bijay Khatri

**Affiliations:** 1 Department of Nephrology, National Academy of Medical Sciences, Kanti Path, Kathmandu, Nepal; 2 Department of Nephrology, Shahid Dharmabhakta National Transplant Centre, Dudhpati, Bhaktapur, Nepal; 3 Academic and Research Department, BP Eye Foundation, Hospital for Children, Eye, ENT, and Rehabilitation Services, Madhyapur Thimi, Bhaktapur, Nepal

**Keywords:** *biopsy*, *hematuria*, *kidney*, *proteinuria*

## Abstract

**Introduction:**

Primary focal segmental glomerulosclerosis is a form of glomerular disease that needs immunosuppressive therapy, which, if untreated, can lead to end-stage renal disease. Ultrastructural analysis by electron microscopy is essential to distinguish primary from other forms of focal segmental glomerulosclerosis. This study aimed to find out the prevalence of primary focal segmental glomerulosclerosis among patients with glomerular diseases undergoing kidney biopsy in a tertiary care centre.

**Methods:**

A descriptive cross-sectional study was done in the Department of Nephrology from 1 January 2022 to 31 December 2022. Data were collected after obtaining ethical approval from the Institutional Review Committee (Reference number: 473/2079/80). The data from clinical and laboratory records of patients with the glomerular disease who underwent kidney biopsy were obtained. Data was collected by using convenience sampling. Point estimate and 95% Confidence Interval were calculated.

**Results:**

Among 213 patients with glomerular disease undergoing kidney biopsy, 22 (10.33%) (6.2414.42, 95% Confidence Interval) were diagnosed with primary focal segmental glomerulosclerosis. All patients had nephrotic range proteinuria, but 2 (9.09%) patients had no features of nephrotic syndrome. Microscopic hematuria was found in 4 (18.18%) patients.

**Conclusions:**

The prevalence of primary focal segmental glomerulosclerosis was lower than in other studies done in similar settings.

## INTRODUCTION

Focal segmental glomerulosclerosis (FSGS) is a histopathologic pattern of podocyte injury with segmental obliteration of the glomerular capillaries in a subset of glomeruli sampled on kidney biopsy.^1,2^ FSGS is a major cause of nephrotic syndrome leading to endstage renal disease.^2,3^ FSGS classification (primary, secondary, adaptive, genetic, or undetermined cause) is used to individualize treatment for medication use, dosage, and duration.

Primary FSGS usually presents with nephrotic syndrome. Other forms, like secondary and maladaptive, usually have nephrotic range proteinuria without serum albumin below 3.5 gm/dl.^4^ Electron microscopy is essential for finding out the podocyte foot process effacement to distinguish primary from other forms, but electron microscopy is not regularly used in Nepal.

This study aimed to find out the prevalence of primary FSGS among patients with glomerular diseases in a tertiary care centre.

## METHODS

A descriptive cross-sectional study was conducted in the Department of Nephrology, National Academy of Medical Sciences. Ethical approval was obtained from the Institutional Review Committee (Reference number: 473/2079/80) of the hospital. The data was collected from 1 January 2022 to 31 December 2022 from the hospital-based record available from the Department of Nephrology, where an e-copy of each light microscopic, immunofluorescence, and electron microscopy test was kept, and the e-copy record was reviewed. Patients whose renal biopsy revealed an absence of glomeruli in either light, immunofluorescence, or electron microscopy were excluded from the study. Data were collected using the convenience sampling technique. The sample size was calculated by using the following formula:


$$\begin{array}{ll} \text{n} & ={\text{Z}}^{2}\times \frac{\text{p}\times \text{q}}{{\text{e}}^{\text{2}}}\\ & =1.96^{2}\times \frac{0.50\times 0.50}{0.07^{2}}\\ & =196 \end{array}$$

Where,

Z = 1.96 at a 95% Confidence Interval (CI)
p = prevalence taken as 50% for maximum sample size calculation
q = 1-p
e = margin of error, 7%

The calculated sample size was 196. However, 213 records were taken for the study.

Kidney biopsy samples of all patients had light and immunofluorescence microscopy. Electron microscopy was sent in 122 patients only. FSGS was diagnosed on light microscopy as having segmental obliteration of glomerular capillaries in a subset of biopsied glomeruli with less than or equal to 1 + staining of any immunoglobulin or complement on immunofluorescence and no other significant lesions.^1^ FSGS was considered primary if podocyte effacement was diffuse (>80%) on electron microscopy.^5^ The histological variants of primary FSGS were categorized as per Columbia classification as a tip, cellular, perihilar, collapsing, and not otherwise specified (NOS) variants.^6^ Nephrotic range proteinuria is defined as a 24-hour urinary protein excretion of ≥3.5 g, and nephrotic syndrome is defined as a 24-hour urinary protein excretion of ≥3.5 g and serum albumin concentration of <3.5 g/dL).^7^ Microscopic hematuria is defined by ≥3 RBCs per high-power field in urine microscopy.^8^

Data were entered and analyzed using Microsoft Excel 2016 and IBM SPSS Statistics version 26.0. Point estimate and 95% CI were calculated.

## RESULTS

Among 213 patients, the prevalence of primary FSGS was 22 (10.33%) (6.24-14.42, 95% CI). According to Columbia Classification, the most common histological variant among categorized primary FSGS was Not Otherwise Specified (NOS) variant 6 (27.27%), no variant was reported in 10 (45.45%) cases (Table 1).

**Table 1:** Histological variants as per Columbia classification in patients with primary FSGS (n= 22).

Variants	n (%)
FSGS collapsing	3 (13.64)
FSGS NOS	6 (27.27)
FSGS tip	3 (13.64)
FSGS uncategorized	10 (45.45)

The mean serum creatinine was 1.41±0.99 mg/dl (Range: 0.62-5.10) and mean serum albumin was 2.42±0.67 gm/dl (Range: 1.50-3.80). Among male patients, mean serum albumin was 2.70±0.72 (Table 2).

**Table 2:** Distribution of serum creatinine and serum albumin in patients with primary FSGS (n= 22).

	Serum creatinine	Serum albumin
	Mean±SD	Mean±SD
Age-group (years)		
16-59	1.17±0.66	2.31±0.78
60-79	1.76±1.31	2.57±0.48
Sex		
Male	1.57±1.24	2.70±0.72
Female	1.24±0.7	2.13±0.5

All patients with primary FSGS had nephrotic range proteinuria. However, 2 (9.09%) patients had no features of nephrotic syndrome. Microscopic hematuria was found in only 4 (18.18%) patients. The mean age of patients was 46.95±20.66 years, and males were 11 (50%) (Table 3).

**Table 3:** Demographic characteristics of patients with primary FSGS (n= 22).

Characteristics	n (%)
**Age-group (years)**	
16-59	13 (59.09)
60-79	9 (40.91)
**Sex**	
Male	11 (50)
Female	11 (50)

## DISCUSSION

Primary FSGS was found in 10.33% of patients with glomerular diseases undergoing renal biopsy in our study. Other studies have suggested that it can occur in up to 19% of native kidney biopsies.^9^ The lower prevalence of primary FSGS in our study might be due to the fact that all patients did not have an electron microscopy study which is essential for diagnosing the condition. Electron microscopy was performed only on 57.28% of the tissue. A possible cause of this might be the cost associated with an additional study by electron microscopy on top of light and immunofluorescence microscopy. The incidence of primary FSGS is generally 1.2-1.5 fold higher in men than in women.^10^ However, our study found equal prevalence in both sex.

Primary FSGS lesions belonging to the NOS type were the most common type of lesion found in our study followed by the tip variant. No category was reported in 12 biopsies and the aggressive collapsing variant was found in three cases. NOS type was the most common type of lesion in primary FSGS followed by tip lesion as in our study.^5^

Patients with primary FSGS have nephrotic syndrome with serum albumin below 3.5 gm/dl besides nephrotic range proteinuria. In our study, 2 (9.09%) patients with nephrotic range proteinuria but without nephrotic syndrome were found to have primary FSGS. A study done in Mayo Clinic showed 1 (4.34%) of the patient had no nephrotic syndrome despite diffuse foot process effacement in FSGS.^5^ The prevalence of nephrotic syndrome in primary FSGS has been reported to vary from 54 to 70% to even 90%.^11-13^ This variation may be due to the inclusion of unrecognized adult-onset genetic FSGS which have diffuse foot process effacement but no frank nephrotic syndrome.

FSGS is a common form of glomerular lesion that needs distinguishing between primary and other forms. Only the primary form of FSGS responds to steroids and other immunosuppressive agents.^14^ Other forms of FSGS need treatment for the respective cause of FSGS or conservative management in the forms of antiproteinuric, antihypertensive, and lipidlowering agents.^15^ Hereditary forms of FSGS require genetic analysis for mutations in genes encoding vital podocyte proteins.

This study was limited to a single centre and can not be generalized. The renal biopsy interpretation was done by different pathologists and inter-observer bias can not be ruled out. Electron microscopy was not feasible in all the patients due to financial constraints which might have underestimated the prevalence of primary FSGS.

## CONCLUSIONS

The prevalence of primary FSGS was lower than in other available studies done in similar settings. The routine use of electron microscopy is bound to distinguish primary from other forms of FSGS and reduce the unnecessary use of immunosuppressive medicines in other forms of FSGS. A larger prospective study on all proteinuric patients using electron microscopy is recommended.

## References

[1] Cattran DC, Feehally J, Cook HT, Cook Terence, Liu Zhi Hong, Fernando C, Fervenza (2012). Kidney disease: improving global outcomes (KDIGO) glomerulonephritis work group. KDIGO clinical practice guideline for glomerulonephritis.. Kidney International Supplements..

[2] Rosenberg AZ, Kopp JB (2017). Focal Segmental Glomerulosclerosis.. Clin J Am Soc Nephrol..

[3] D Agati VD, Kaskel FJ, Falk RJ (2011). Focal segmental glomerulosclerosis.. N Engl J Med..

[4] De Vriese AS, Sethi S, Nath KA, Glassock RJ, Fervenza FC (2018). Differentiating primary, genetic, and secondary FSGS in adults: A clinicopathologic approach.. J Am Soc Nephrol..

[5] Sethi S, Zand L, Nasr SH, Glassock RJ, Fervenza FC (2014). Focal and segmental glomerulosclerosis: clinical and kidney biopsy correlations.. Clin Kidney J..

[6] D Agati VD, Fogo AB, Bruijn JA, Jennette JC (2004). Pathologic classification of focal segmental glomerulosclerosis: a working proposal.. Am J Kidney Dis..

[7] Glassock RJ, Fervenza FC, Hebert L, Cameron JS (2015). Nephrotic syndrome redux.. Nephrol Dial Transplant..

[8] Barocas DA, Boorjian SA, Alvarez RD, Downs TM, Gross CP, Hamilton BD (2020). Microhematuria: AUA/SUFU Guideline.. J Urol..

[9] D Agati V (1994). The many masks of focal segmental glomerulosclerosis.. Kidney Int..

[10] O Shaughnessy MM, Hogan SL, Thompson BD, Coppo R, Fogo AB, Jennette JC (2018). Glomerular disease frequencies by race, sex and region: results from the International Kidney Biopsy Survey.. Nephrol Dial Transplant..

[11] Kambham N, Markowitz GS, Valeri AM, Lin J, D Agati VD (2001). Obesity-related glomerulopathy: an emerging epidemic.. Kidney Int..

[12] Thomas DB, Franceschini N, Hogan SL, Ten Holder S, Jennette CE, Falk RJ (2006). Clinical and pathologic characteristics of focal segmental glomerulosclerosis pathologic variants.. Kidney Int..

[13] Praga M, Morales E, Herrero JC, Perez Campos A, Dominguez-Gil B (1999). Absence of hypoalbuminemia despite massive proteinuria in focal segmental glomerulosclerosis secondary to hyperfiltration.. Am J Kidney Dis..

[14] Allard L, Kwon T, Krid S, Bacchetta J, Garnier A, Novo R (2018). Treatment by immunoadsorption for recurrent focal segmental glomerulosclerosis after paediatric kidney transplantation: a multicentre French cohort.. Nephrol Dial Transplant..

[15] D Agati VD (2017). Podocyte growing pains in adaptive FSGS.. J Am Soc Nephrol..

